# Lifestyle clusters and bone mineral density in Chinese adults: a cross-sectional study with cluster analysis

**DOI:** 10.3389/fendo.2026.1904158

**Published:** 2026-09-04

**Authors:** Senyang Lei, Manlin Wang, Xudan Lei, Zhen Lei

**Affiliations:** 1 Outpatient Department, Air Force Medical Center, PLA, Beijing, China; 2 Beijing National Day School, Beijing, China; 3 School of Computer and Cyber Sciences, Communication University of China, Beijing, China; 4 Basic Courses Department, Space Engineering University, Beijing, China

**Keywords:** bone mineral density, calcium supplementation behavior, lifestyle clustering, osteoporosis, physical activity

## Abstract

**Background:**

Osteoporosis is a major global public health challenge, with lifestyle as a key modifiable factor. This study aimed to examine associations between lifestyle patterns and bone mineral density (BMD) in Chinese adults.

**Methods:**

In this cross-sectional, multi-center study, 5,324 adults from Beijing (2016–2020) with complete lifestyle questionnaires were analyzed (lumbar spine BMD available for 4,028; hip BMD available for 4,654). Each of eight lifestyle variables was residualized on age and sex before k-means clustering (k = 3) categorized participants into three groups: office/mental-labor workers with low leisure-time activity (OL), physical-labor workers (PL), and office/mental-labor workers with an active leisure-time lifestyle (OA). Differences in lumbar spine and hip BMD among clusters were examined using multivariable-adjusted models (age modeled via restricted cubic spline, sex, body mass index [BMI], hospital fixed effects), with stratification by sex, age group, and BMI group, alongside full-cohort dose-response and cluster × exposure interaction analyses.

**Results:**

After adjustment, the OA cluster showed higher lumbar spine BMD than the OL cluster (adjusted difference = 0.024 g/cm^2^, 95% CI 0.012–0.036, *P* < 0.001). The PL cluster showed lower hip BMD than both the OL cluster (adjusted difference = −0.041 g/cm^2^, 95% CI −0.056 to −0.027, *P* < 0.001) and the OA cluster (adjusted difference = 0.028 g/cm^2^, 95% CI 0.011–0.044, *P* = 0.002), despite the occupational physical activity inherent to manual labor. These associations were broadly consistent across sex, age, and BMI strata, though attenuated among participants aged ≥70 years and those with obesity. In dose-response analyses, exercise and sun exposure showed non-linear associations with BMD at both sites, while calcium supplementation frequency showed a consistent negative linear association that did not vary by cluster.

**Conclusion:**

A lifestyle pattern combining active leisure-time behavior with office-based occupation was associated with higher lumbar spine BMD, while an occupationally physical-labor lifestyle was associated with lower hip BMD relative to both other clusters. These associations exhibited site-specific patterns and were generally consistent across demographic subgroups. Given the cross-sectional design, these findings should be interpreted as associations rather than causal effects; prospective studies are needed to clarify their causal structure and clinical significance.

## 1 Introduction

Osteoporosis is a systemic bone disease characterized by low bone mineral density (BMD), deterioration of bone micro-architecture and impaired bone strength, which significantly increases the risk of fragility fractures (1). Epidemiological data shows that there were approximately 41.5 million osteoporosis patients worldwide in 2019, and it is expected that the total number of patients will reach 263.2 million between 2030 and 2034 (2). The most direct and serious consequence of osteoporosis is an increased risk of fractures: in the 50 years old population, about half of women and one-third of men will experience at least one osteoporotic fracture during their remaining lifetime (3). Such fractures not only seriously affect quality of life but also frequently lead to hospitalization, long-term disability and even death, posing a particular threat to high-risk groups such as postmenopausal women and those with other chronic diseases (such as cancer) (4, 5). With the acceleration of global aging, combined with factors such as changes in dietary habits, reduced physical activity, and increased sedentary behavior in modern lifestyles, the prevalence of osteoporosis is expected to further increase in the future (1, 2, 6). Therefore, promoting healthy lifestyles has become a key strategy for preventing osteoporosis.

In China, osteoporosis also poses a major public health challenge, making the exploration of effective prevention and management strategies a critical focus. The risk factors for osteoporosis can be divided into two major categories: non-modifiable factors and modifiable factors. The former includes race, age, sex, menopausal status (particularly premature menopause), personal history of fracture, and genetic background (e.g., vitamin D receptor gene polymorphisms). The latter encompasses lifestyle and dietary factors such as physical activity levels, body weight, nutritional status, sunlight exposure, thyroid function and alcohol and tobacco use (7–9). However, a systematic understanding of how modifiable lifestyle factors collectively influence bone density—specifically, a comprehensive multifactorial model—is still lacking.

## 2 Methods

### 2.1 Participants

**Figure 1:**
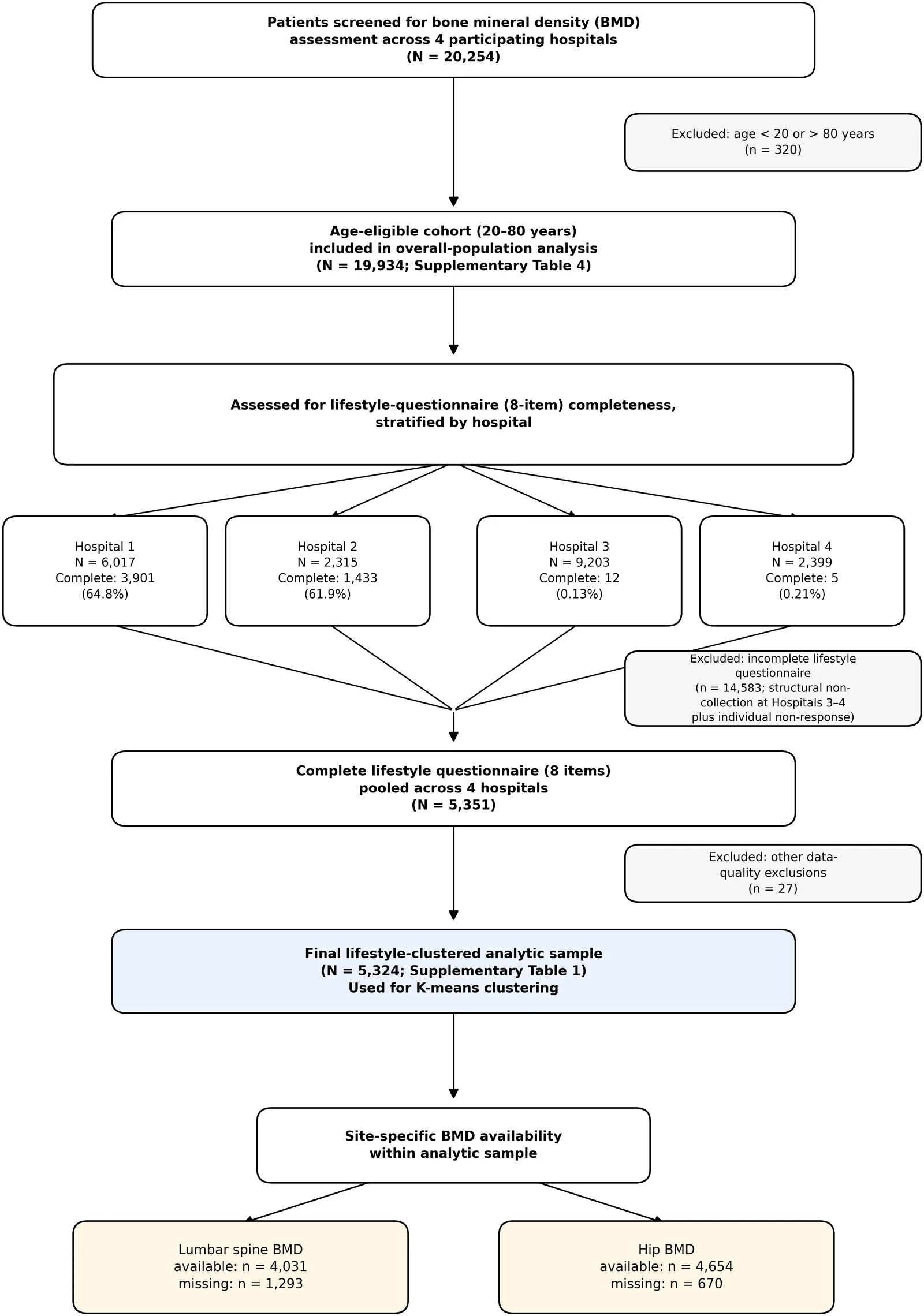
Participant flow diagram. Of 20,254 participants screened across four participating hospitals, 320 were excluded for age <20 or ≥80 years, yielding 19,934 age-eligible participants (overall population). Lifestyle-questionnaire completion rates differed markedly by hospital (64.8% and 61.9% at two hospitals vs. 0.13% and 0.21% at the remaining two), reflecting hospital-level structural non-collection rather than random individual non-response. After excluding 14,583 participants with incomplete lifestyle-questionnaire data, 5,351 participants had complete data; a further 27 were excluded for other data-quality reasons (17 from the two hospitals with near-complete non-collection, whose small number of “complete” records did not proceed to final analysis, and 10 from the remaining two hospitals), yielding the final lifestyle-clustered analytic sample (N = 5,324), which was used for lifestyle clustering and all cluster-based BMD analyses. Within this analytic sample, lumbar spine BMD was available for 4,028 participants and hip BMD for 4,654 participants.

This multicenter, cross-sectional study recruited participants from four hospitals in Beijing. All participants underwent routine annual health examinations and dual-energy X-ray absorptiometry (DXA) scans. Inclusion criteria were: (1) age between 20 and 79 years; (2) completion of all questionnaires and the DXA scans. Exclusion criteria were: (1) pregnancy or lactation; (2) history of major metabolic bone diseases (e.g., hyperparathyroidism, multiple myeloma, renal osteodystrophy); (3) use of glucocorticoids, anti-osteoporosis medications, or other bone metabolism-affecting hormones within the past six months; (4) presence of metal implants or history of contrast agent use that may interfere with DXA results; (5) malignancies with bone metastasis or skeletal involvement such as myeloma; (6) prior orthopedic surgery involving the lumbar spine or hip (e.g., joint replacement, spinal fusion); (7) recent radionuclide imaging or barium contrast studies within one month that could affect DXA interpretation; (8) non-Chinese nationality. Participant recruitment and the derivation of the analytic samples are summarized in **Figure 1**. Among the 19,934 age-eligible participants, lifestyle-questionnaire completion rates differed markedly across the four hospitals (64.8% and 61.9% at two hospitals vs. 0.13% and 0.21% at the remaining two), indicating hospital-level structural non-collection as the predominant source of missingness. The four participating centers comprised one lead institution (Air Force Medical Center, PLA) and three of its affiliated health-examination centers, operating under shared institutional ethics oversight. This study conformed to the standards set by the Declaration of Helsinki, and was approved by the Institutional Review Board of the Air Force Medical Center, PLA (approval number: 空特(科研)第2019-31-PJ01).

### 2.2 Procedure

Participants completed standardized questionnaires and underwent physical examinations. Sex, age, height, weight, and body mass index (BMI) were recorded. Examinations at each participating hospital were performed by a dedicated, experienced technician at that site. Hologic Horizon-Wi devices were used at Hospitals 1, 2, and 3, and GE Lunar iDXA was used at Hospital 4. Formal cross-calibration between devices/hospitals was not performed.

A panel of six experts conducted a two-round Delphi survey (10) to evaluate and refine the initial questionnaire. In the first round, based on expert ratings and suggestions, five items were deleted, six items were merged, and two new items were added. The second round focused on structural optimization and logical flow adjustments, resulting in a final questionnaire comprising 10 core variables across five domains. All data were encrypted and uploaded in real-time to the “Chinese Population Bone Density Database”. A double-data entry process with logical validation was implemented to ensure data completeness and accuracy. Response options and category definitions for the eight lifestyle items are provided in **Supplementary Table 1**.

### 2.3 Endpoint

The primary endpoint was the difference in mean BMD at the lumbar spine and hip across distinct lifestyle clusters within the overall sample. Secondary endpoints included: 1) comparisons of cluster-based BMD differences according to four stratification schemes: sex, age group, sex × age group interaction, and BMI; 2) cluster × exposure interaction effects for three modifiable factors (sun exposure, physical exercise, and calcium supplementation) on lumbar/hip BMD, estimated in the full cohort.

### 2.4 Statistical analysis

The overall, non-stratified comparison of BMD across lifestyle clusters (adjusted for age, sex, BMI, and hospital) was prespecified as the primary analysis, with Bonferroni correction applied to the three pairwise cluster comparisons within each skeletal site. All stratified comparisons (by sex, age group, sex × age group, and BMI group), dose-response analyses, and cluster × exposure interaction analyses were considered secondary and exploratory in nature; no single combined multiplicity correction was applied across this broader set of analyses, and effect estimates with 95% confidence intervals are emphasized throughout in place of isolated *P*-value thresholds.

The eight lifestyle variables were first residualized on age and sex to remove demographic confounding prior to clustering: binary variables (self-rated health, occupation type, smoking) via logistic regression, with the residual defined as the observed value minus the model-predicted probability; ordinal variables with more than two levels (sun exposure, exercise, alcohol consumption, beverage preference, calcium supplementation) via proportional-odds ordinal logistic regression, with the residual defined as the observed category minus the model-implied expected category value. The eight resulting residuals were standardized (z-scored) and clustered using k-means (Euclidean distance) with an optimized multi-start procedure (50 k-means++ initializations, best solution retained by inertia). Cluster number (k) was selected by comparing silhouette width and Calinski-Harabasz index across k = 2–6 (**Supplementary Figure 1**); the k = 3 solution was verified stable across 5 independent optimized searches (pairwise Adjusted Rand Index ≥ 0.99) and preferred over k = 4 following a bootstrap comparison showing no reliable difference in fit (full justification in **Supplementary Text 1**; stability verification, and comparison with the original grouping in **Supplementary Table 2**). The between-cluster explanatory power (η^2^) of each of the eight lifestyle variables is reported in **Supplementary Table 3**, indicating that calcium supplementation contributed negligibly to cluster definition (η^2^ = 0.0006, *P* = 0.195), while occupation type, sun exposure, beverage preference, and self-rated health were the dominant contributors. Baseline characteristics and BMD were summarized as mean ± standard deviation (SD) or number (percentage). Differences among clusters were assessed using one-way analysis of variance (ANOVA). If the overall F-test yielded a *P* < 0.05, *post-hoc* pairwise comparisons with Bonferroni correction were performed, and the adjusted *p*-values were reported. Implausible BMD values were identified using a site-specific threshold of |z-score| > 3 SDs from the mean. As a sensitivity analysis, we additionally re-ran the primary analysis of covariance (ANCOVA) model after excluding these observations and compared the resulting estimates against the primary analysis (which retained the full sample).

Stratified analyses were conducted by repeating the ANOVA procedure according to the following four schemes: (1) sex; (2) age group (<50, 50–59, 60–69, 70–79 years); (3) sex × age group interaction strata; and (4) BMI tertiles (Participants were categorized into three BMI groups based on the Chinese population-specific classification: normal weight (<24 kg/m^2^), overweight (24–27.9 kg/m^2^), and obese (≥28 kg/m^2^). Within each stratum, the mean ± SD of BMD for each cluster and the *p*-values for inter-cluster differences were reported. To assess the primary cluster-BMD association while adjusting for confounding, we additionally fit multivariable linear regression models with BMD as the outcome, lifestyle cluster as the primary predictor, and age (modeled via a restricted cubic spline with 4 knots), sex, BMI, and hospital (fixed effect) as covariates. Adjusted marginal means and pairwise adjusted mean differences with 95% confidence intervals were derived from these models.

Multiple linear regression models were employed to examine the associations between modifiable factors and BMD. Dose-response relationships between each lifestyle exposure (sun exposure, exercise, calcium supplementation) and BMD were examined in the full analytic cohort using category-level (non-linear) modeling to test for departures from linearity, adjusted for age, sex, BMI, and hospital. All statistical analyses were performed using Stata 17.0, and a two-sided *P* ≤ 0.05 was considered statistically significant.

## 3 Results

### 3.1 Baseline demographic characteristics

From July 2016 through December 2020, a total of 19,934 participants were included in the study. The overall population had a mean age of 54.5 ± 14.5 years, and 9,865 males (49.5%) (**Supplementary Table 4**). Among these 19,934 age-eligible participants, lifestyle-questionnaire completion rates differed markedly across the four hospitals (64.8% and 61.9% at two hospitals vs. 0.13% and 0.21% at the remaining two), and 5,324 participants with complete lifestyle data were included in the clustering analysis (**Figure 1**). Compared with the overall age-eligible population, the analytic sample was comparable in BMI and hip BMD, but was significantly older and included a higher proportion of women (61.7% vs. 50.5%), likely reflecting between-hospital differences in patient case-mix that also underlie the differential questionnaire completion rates (**Supplementary Table 5**). **Supplementary Table 1** presents the distribution of the eight lifestyle variables in the clustered sample.

### 3.2 Profiles of each cluster

The silhouette width and Calinski-Harabasz index for the primary k = 3 solution were 0.309 and 881.1, respectively (**Supplementary Figure 1**), exceeding the conventional threshold for meaningful cluster structure. The three resulting clusters were labeled descriptively based on their profiles across the eight lifestyle variables (**Supplementary Table 2**).

Cluster 1, the “Office-Low-Activity” (OL) group (n = 3,803, 71.5%), was the most prevalent cluster and was composed entirely of participants engaged in mental/office-based occupations. This group reported the poorest self-rated health (98.6% rated their health as average rather than good), the lowest daylight exposure (80.6% reported less than 30 minutes of daily sun exposure, and only 0.7% reported more than 60 minutes), and limited vigorous exercise (only 2.3% exercised more than 60 minutes daily, although 43.0% reported 30–60 minutes). Smoking, alcohol consumption, and calcium supplementation were all relatively infrequent in this group. This profile represents a predominantly sedentary, office-based lifestyle with limited outdoor and leisure-time physical activity.

Cluster 2, the “Physical-Labor” (PL) group (n = 453, 8.5%), was the smallest cluster and was defined entirely by occupation: all members were engaged in manual/physical labor. Reflecting the nature of their occupation, this group reported the highest daylight exposure (41.1% reported more than 60 minutes daily) and relatively high exercise levels (36.2% reported more than 60 minutes daily), alongside better self-rated health than the OL group (20.8% rated their health as good). This group also had the highest rate of regular calcium supplementation (18.8%). This profile represents an occupationally active lifestyle in which physical activity and outdoor exposure are largely incidental to manual work rather than deliberate leisure-time behavior.

Cluster 3, the “Office-Active” (OA) group (n = 1,068, 20.0%), comprised office/mental-labor workers who nonetheless reported the most health-promoting leisure-time behaviors among the three groups: the best self-rated health (39.2% rated their health as good, the highest of the three clusters), the highest exercise levels (44.3% reported more than 60 minutes daily, exceeding even the PL group), and the highest daylight exposure (42.6% reported more than 60 minutes daily). This profile represents office-based workers who actively engage in leisure-time exercise and outdoor activity to compensate for an otherwise sedentary occupation.

### 3.3 Age-related trends in bone mineral density

**Figure 2:**
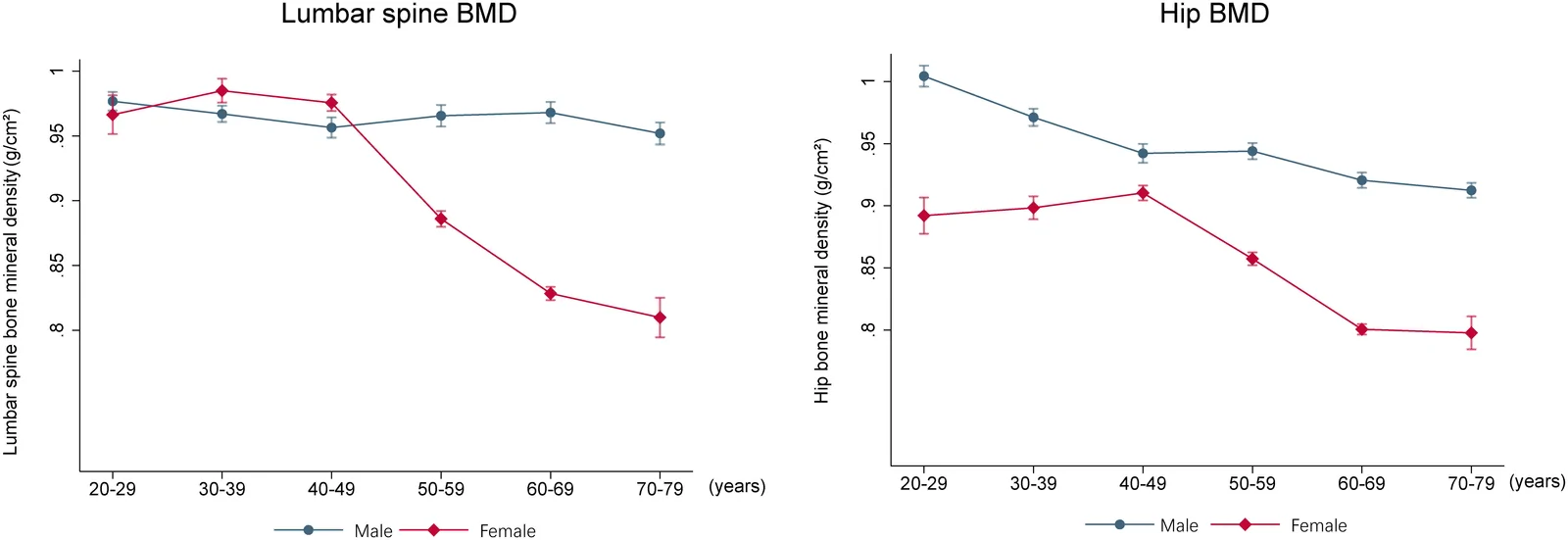
Age- and sex-stratified trends in lumbar spine and hip bone mineral density (BMD). Mean BMD (g/cm^2^) is plotted by 10-year age group (20–29, 30–39, 40–49, 50–59, 60–69, 70–79 years; participants aged <20 or ≥80 years excluded) for men (circles, blue) and women (diamonds, red), using unadjusted (raw) values from the overall age-eligible population (N = 19,934; men, n = 9,865; women, n = 10,069; per-group-per-sex sample sizes range from n = 235 to n = 3,557). Error bars represent 95% confidence intervals (mean ± 1.96 × standard error). No formal statistical comparison is annotated on the figure; age- and sex-related patterns are described qualitatively in the Results.

**Figure 2** shows an age-related decline in hip and lumbar BMD in both sexes, with women consistently lower than men. Hip BMD peaked at 1.00 g/cm^2^ in men aged 20-29, falling to 0.91 g/cm^2^ by 70-79. In women, hip BMD peaked later (0.91 g/cm^2^ at 40-49) and declined more sharply to 0.80 g/cm^2^ at 70-79, a total drop of ~12%. Lumbar BMD followed a similar pattern, with accelerated decline in women after age 50. By 60–69 years, lumbar BMD in women (0.83 g/cm^2^) was significantly lower than that in men (0.97 g/cm^2^). A marked stepwise decrease occurred in women around age 50, coinciding with menopause.

### 3.4 Associations between lifestyle clusters and bone mineral density

**Figure 3:**
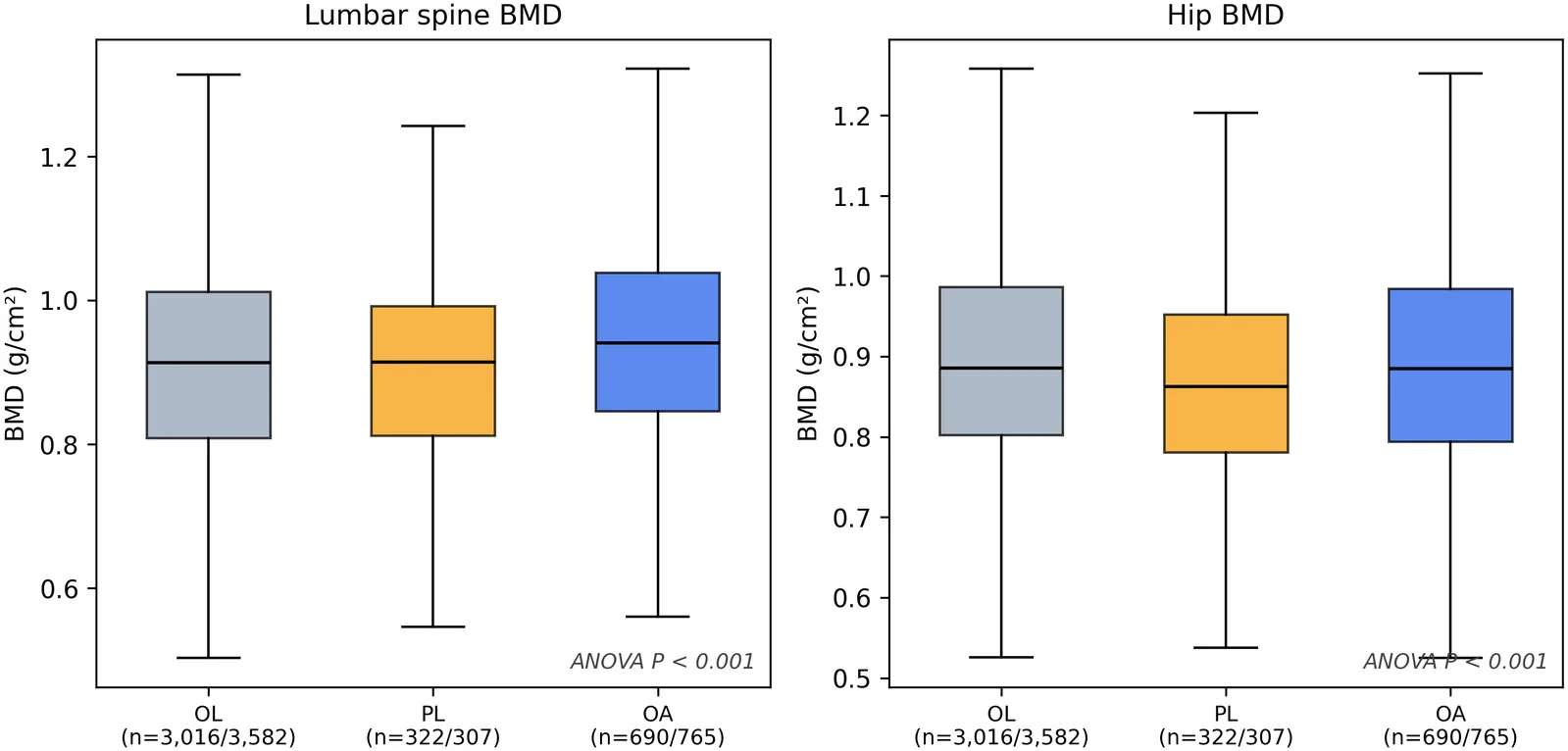
Lumbar spine and hip bone mineral density (BMD) by lifestyle cluster. Box plots show the median (center line), interquartile range (box), and 1.5×IQR whiskers, for unadjusted BMD values from the analytic sample (N = 5,324), by cluster: OL (office/mental-labor workers with low leisure-time activity; lumbar spine n = 3,016, hip n = 3,582), PL (physical-labor workers; lumbar spine n = 322, hip n = 307), OA (office/mental-labor workers with an active leisure-time lifestyle; lumbar spine n = 690, hip n = 765). *P*-values are from one-way ANOVA on unadjusted values; adjusted comparisons are reported in **Table 1**.

In unadjusted comparisons, significant differences in lumbar spine and hip BMD were observed among the three lifestyle clusters (overall ANOVA *P* < 0.001 at both sites; **Supplementary Table 6**; **Figure 3**). The OA cluster showed the highest unadjusted lumbar spine BMD (0.946 ± 0.152 g/cm^2^), significantly exceeding the OL cluster (difference = 0.032 g/cm^2^, Bonferroni-corrected *P* < 0.001); the PL cluster showed the lowest unadjusted hip BMD (0.862 ± 0.135 g/cm^2^), significantly lower than both the OL (difference = −0.034 g/cm^2^, *P* < 0.001) and OA clusters (difference = 0.029 g/cm^2^, *P* = 0.007).

After adjusting for age (modeled via a restricted cubic spline), sex, BMI, and hospital in the primary ANCOVA model (**Table 1**), these associations remained materially unchanged in magnitude. The OA cluster retained significantly higher adjusted lumbar spine BMD than the OL cluster (adjusted difference = 0.024 g/cm^2^, 95% CI 0.012–0.036, *P* < 0.001), while the difference between OA and PL was not statistically significant (adjusted difference = 0.018, 95% CI −0.002–0.037, *P* = 0.218). For hip BMD, the PL cluster showed significantly lower adjusted BMD than both the OL cluster (adjusted difference = −0.041, 95% CI −0.056 to −0.027, *P* < 0.001) and the OA cluster (adjusted difference = 0.028, 95% CI 0.011–0.044, *P* = 0.002); the difference between OA and OL was smaller but remained significant (adjusted difference = −0.014, 95% CI −0.023 to −0.004, *P* = 0.015). Age showed a significant departure from linearity at both skeletal sites (*P* < 0.001), supporting the use of the flexible age specification. These adjusted results indicate that the observed cluster-BMD associations are not primarily attributable to age, sex, BMI, or hospital-level confounding.

**Table 1:** ANCOVA-adjusted BMD comparison across lifestyle clusters (age modeled via restricted cubic spline).

Site	Cluster	N	Unadjusted mean ± SD	Adjusted mean (95% CI)^a^	Pairwise comparison	Adjusted difference (95% CI)	Bonferroni- adjusted *P*^b^
Lumbar spine	OL	3016	0.914 ± 0.155	0.916 (0.910, 0.921)	PL vs OL	0.006 (-0.010, 0.023)	1.000
	PL	322	0.925 ± 0.203	0.922 (0.906, 0.938)	OA vs OL	0.024 (0.012, 0.036)	**<0.001**
	OA	690	0.945 ± 0.152	0.940 (0.929, 0.950)	OA vs PL	0.018 (-0.002, 0.037)	0.218
Hip	OL	3582	0.896 ± 0.143	0.898 (0.894, 0.902)	PL vs OL	-0.041 (-0.056, -0.027)	**<0.001**
	PL	307	0.862 ± 0.135	0.856 (0.843, 0.870)	OA vs OL	-0.014 (-0.023, -0.004)	**0.015**
	OA	765	0.891 ± 0.138	0.884 (0.875, 0.893)	OA vs PL	0.028 (0.011, 0.044)	**0.002**

Clusters derived from k-means (k = 3) applied to the eight lifestyle variables after residualizing each on age and sex (logistic regression for binary items; ordinal logistic regression for ordinal items), standardized prior to clustering; solution verified stable across 5 independent multi-start searches (pairwise Adjusted Rand Index ≥ 0.99). OL, office/mental-labor workers with low leisure-time activity (n = 3,803, 71.5%); PL, physical-labor workers (n = 453, 8.5%); OA, office/mental-labor workers with an active leisure-time lifestyle (n = 1,068, 20.0%). Model: BMD ~ Cluster + restricted cubic spline of age (4 knots) + sex + BMI + hospital (fixed effect), fit in the full lifestyle-clustered analytic sample (N = 5,324; site-specific N varies by BMD availability). Age showed a significant departure from linearity at both skeletal sites (P < 0.001, nested F-test comparing spline vs. linear age specification), supporting the flexible age specification.

Bold values indicate statistical significance.

^a^Adjusted means are predictive margins (average model-based predictions across the observed covariate distribution).

^b^Bonferroni correction factor = 3 (three pairwise comparisons per site); adjusted *P* < 0.05 considered statistically significant. Unadjusted means shown for direct comparison. BMD, bone mineral density; CI, confidence interval; SD, standard deviation.

A sensitivity analysis excluding 16 lumbar spine and 26 hip observations with implausible values (|z-score| > 3) yielded essentially unchanged estimates (lumbar spine OA-vs-OL adjusted difference: 0.024 vs. 0.022 g/cm^2^; hip PL-vs-OL adjusted difference: −0.041 vs. −0.039 g/cm^2^; both *P* < 0.001 before and after exclusion), confirming that the primary findings are not driven by these observations (results not tabulated).

### 3.5 Stratified analyses of lifestyle clusters and BMD

The following stratified analyses are exploratory and hypothesis-generating in nature.

#### 3.5.1 Sex stratification

Among women, the OA cluster showed significantly higher lumbar spine BMD than both the OL cluster (difference = 0.031 g/cm^2^, Bonferroni *P* < 0.001) and the PL cluster (difference = 0.035, *P* = 0.019); hip BMD was significantly lower in the PL cluster than the OL cluster (difference = −0.033, *P* = 0.003). Among men, hip BMD followed a similar pattern, with the PL cluster significantly lower than the OL cluster (difference = −0.043, *P* = 0.003); lumbar spine differences among men did not reach significance after Bonferroni correction (**Table 2**).

**Table 2:** Exploratory sex-stratified BMD comparison across clusters.

Site	Sex	Cluster	N	Mean ± SD (g/cm^2^)	*P*-value	Pairwise	Difference (g/cm^2^)^a^	Adjusted *P*^b^
Lumbar	Male	OL	1146	0.968 ± 0.146	**0.012**	OL vs PL	-0.032	0.093
		PL	125	1.000 ± 0.258		OL vs OA	-0.024	0.056
		OA	291	0.992 ± 0.149		PL vs OA	+0.008	1.000
Lumbar	Female	OL	1870	0.881 ± 0.150	**<0.001**	OL vs PL	+0.004	1.000
		PL	198	0.877 ± 0.139		OL vs OA	-0.031	**<0.001**
		OA	401	0.912 ± 0.145		PL vs OA	-0.035	**0.019**
Hip	Male	OL	1344	0.959 ± 0.138	**0.002**	OL vs PL	+0.043	**0.003**
		PL	126	0.916 ± 0.130		OL vs OA	+0.013	0.349
		OA	344	0.946 ± 0.137		PL vs OA	-0.030	0.112
Hip	Female	OL	2238	0.858 ± 0.132	**0.002**	OL vs PL	+0.033	**0.003**
		PL	181	0.824 ± 0.125		OL vs OA	+0.011	0.331
		OA	421	0.847 ± 0.122		PL vs OA	-0.022	0.162

Clusters derived from k-means (k = 3) on age/sex-residualized lifestyle variables (see **Supplementary Table 2**).

^a^Difference = Mean (Group1) – Mean (Group2).

^b^Using Bonferroni correction (correction factor=3).

Bold values indicate statistical significance.

OL, office/mental-labor workers with low leisure-time activity; PL, physical-labor workers; OA, office/mental-labor workers with an active leisure-time lifestyle. *P*-value from one-way ANOVA within each stratum; pairwise comparisons via Bonferroni-corrected *post-hoc* tests (correction factor = 3). SD, standard deviation.

#### 3.5.2 Age stratification

In the <50 age group, the OA cluster showed significantly higher lumbar spine BMD than the OL cluster (difference = 0.027 g/cm^2^, *P* = 0.010). In the 50–59 and 60–69 age groups, hip BMD was consistently lower in the PL cluster than the OL cluster (differences of −0.034 and −0.050 g/cm^2^, *P* = 0.025 and *P* < 0.001, respectively). In the ≥70 age group, no significant pairwise differences were observed among the three clusters at either skeletal site (all Bonferroni-corrected *P* > 0.35) (**Table 3**).

**Table 3:** Exploratory age-stratified BMD comparison across clusters.

Site	Age group	Cluster	N	Mean ± SD (g/cm^2^)	*P*-value	Pairwise	Difference (g/cm^2^)^a^	Adjusted *P*^b^
Lumbar	<50	OL	776	0.959 ± 0.131	**0.012**	OL vs PL	-0.013	1.000
		PL	80	0.972 ± 0.124		OL vs OA	-0.027	**0.010**
		OA	255	0.986 ± 0.125		PL vs OA	-0.014	1.000
Lumbar	50-59	OL	970	0.913 ± 0.149	**0.067**	OL vs PL	-0.012	1.000
		PL	111	0.925 ± 0.260		OL vs OA	-0.029	0.066
		OA	200	0.942 ± 0.163		PL vs OA	-0.017	1.000
Lumbar	60-69	OL	1215	0.888 ± 0.165	**0.516**	OL vs PL	-0.006	1.000
		PL	116	0.894 ± 0.151		OL vs OA	-0.014	0.790
		OA	199	0.902 ± 0.161		PL vs OA	-0.008	1.000
Lumbar	≥70	OL	55	0.872 ± 0.164	**0.422**	OL vs PL	-0.036	1.000
		PL	16	0.908 ± 0.339		OL vs OA	-0.052	0.601
		OA	38	0.924 ± 0.142		PL vs OA	-0.016	1.000
Hip	<50	OL	808	0.954 ± 0.141	**0.594**	OL vs PL	+0.012	1.000
		PL	71	0.942 ± 0.124		OL vs OA	+0.009	1.000
		OA	237	0.945 ± 0.126		PL vs OA	-0.003	1.000
Hip	50-59	OL	1061	0.906 ± 0.133	**0.028**	OL vs PL	+0.034	**0.025**
		PL	109	0.872 ± 0.111		OL vs OA	+0.007	1.000
		OA	230	0.899 ± 0.129		PL vs OA	-0.027	0.233
Hip	60-69	OL	1611	0.867 ± 0.137	**<0.001**	OL vs PL	+0.050	**<0.001**
		PL	105	0.817 ± 0.126		OL vs OA	+0.015	0.415
		OA	215	0.852 ± 0.134		PL vs OA	-0.035	0.092
Hip	≥70	OL	102	0.785 ± 0.145	**0.211**	OL vs PL	+0.012	1.000
		PL	22	0.773 ± 0.177		OL vs OA	-0.035	0.359
		OA	83	0.820 ± 0.147		PL vs OA	-0.047	0.573

Clusters derived from k-means (k = 3) on age/sex-residualized lifestyle variables (see **Supplementary Table 2**).

^a^Difference = Mean (Group1) – Mean (Group2).

^b^Using Bonferroni correction (correction factor=3).

Bold values indicate statistical significance.

OL, office/mental-labor workers with low leisure-time activity; PL, physical-labor workers; OA, office/mental-labor workers with an active leisure-time lifestyle. *P*-value from one-way ANOVA within each stratum; pairwise comparisons via Bonferroni-corrected *post-hoc* tests (correction factor = 3). SD, standard deviation.

#### 3.5.3 Sex × age stratification

Patterns of association were generally consistent with the overall and single-stratified results but attenuated in statistical significance within these smaller subgroups, as expected given the reduced sample size per cell. Among men aged 50–59, hip BMD was significantly lower in the PL cluster than in the OL cluster (difference = −0.068 g/cm^2^, *P* = 0.003); a similar pattern was observed among men aged 60–69 (difference = −0.069, *P* = 0.012) and women aged 60–69 (difference = −0.038, *P* = 0.038). Among women aged ≥70, no significant difference was observed for hip BMD; however, a significant pairwise difference in lumbar spine BMD remained between the PL and OA clusters in this subgroup (difference = 0.132 g/cm^2^, Bonferroni-corrected *P* = 0.017), suggesting that lifestyle-related differences in lumbar BMD may persist into advanced age even as hip-BMD differences attenuate. Among men aged ≥70, no pairwise comparisons reached statistical significance after Bonferroni correction (**Table 4**).

**Table 4:** Exploratory age × sex-stratified BMD comparison across clusters.

Site	Sex, age group	Cluster	N	Mean ± SD (g/cm^2^)	*P*-value	Pairwise	Difference (g/cm^2^)^a^	Adjusted *P*^b^
Lumbar	Male, <50	OL	376	0.957 ± 0.127	**0.086**	OL vs PL	-0.026	0.600
		PL	45	0.983 ± 0.135		OL vs OA	-0.026	0.138
		OA	131	0.983 ± 0.121		PL vs OA	0.000	1.000
Lumbar	Male, 50-59	OL	346	0.967 ± 0.144	**0.060**	OL vs PL	-0.051	0.326
		PL	37	1.018 ± 0.391		OL vs OA	-0.046	0.158
		OA	72	1.013 ± 0.179		PL vs OA	+0.005	1.000
Lumbar	Male, 60-69	OL	410	0.976 ± 0.163	**0.880**	OL vs PL	+0.006	1.000
		PL	38	0.970 ± 0.148		OL vs OA	-0.008	1.000
		OA	77	0.984 ± 0.163		PL vs OA	-0.014	1.000
Lumbar	Male, ≥70	OL	14	1.010 ± 0.163	**0.112**	OL vs PL	-0.238	0.143
		PL	5	1.248 ± 0.446		OL vs OA	-0.008	1.000
		OA	11	1.018 ± 0.127		PL vs OA	+0.230	0.188
Lumbar	Female, <50	OL	400	0.960 ± 0.135	**0.084**	OL vs PL	+0.003	1.000
		PL	35	0.957 ± 0.109		OL vs OA	-0.030	0.087
		OA	124	0.990 ± 0.129		PL vs OA	-0.033	0.595
Lumbar	Female, 50-59	OL	624	0.882 ± 0.144	**0.340**	OL vs PL	+0.004	1.000
		PL	74	0.878 ± 0.140		OL vs OA	-0.020	0.484
		OA	128	0.902 ± 0.138		PL vs OA	-0.024	0.760
Lumbar	Female, 60-69	OL	805	0.843 ± 0.148	**0.663**	OL vs PL	-0.014	1.000
		PL	78	0.857 ± 0.139		OL vs OA	-0.007	1.000
		OA	122	0.850 ± 0.136		PL vs OA	+0.007	1.000
Lumbar	Female, ≥70	OL	41	0.825 ± 0.137	**0.016**	OL vs PL	+0.071	0.333
		PL	11	0.754 ± 0.091		OL vs OA	-0.061	0.180
		OA	27	0.886 ± 0.132		PL vs OA	-0.132	**0.017**
Hip	Male, <50	OL	418	0.981 ± 0.147	**0.940**	OL vs PL	+0.008	1.000
		PL	42	0.973 ± 0.132		OL vs OA	+0.001	1.000
		OA	133	0.980 ± 0.128		PL vs OA	-0.007	1.000
Hip	Male, 50-59	OL	367	0.967 ± 0.129	**<0.001**	OL vs PL	+0.068	**0.003**
		PL	46	0.899 ± 0.098		OL vs OA	+0.038	**0.031**
		OA	99	0.929 ± 0.147		PL vs OA	-0.030	0.632
Hip	Male, 60-69	OL	530	0.940 ± 0.131	**0.008**	OL vs PL	+0.069	**0.012**
		PL	32	0.871 ± 0.151		OL vs OA	+0.023	0.435
		OA	83	0.917 ± 0.125		PL vs OA	-0.046	0.271
Hip	Male, ≥70	OL	29	0.880 ± 0.138	**0.439**	OL vs PL	-0.006	1.000
		PL	6	0.886 ± 0.103		OL vs OA	-0.047	0.640
		OA	29	0.927 ± 0.149		PL vs OA	-0.041	1.000
Hip	Female, <50	OL	390	0.924 ± 0.128	**0.132**	OL vs PL	+0.027	0.764
		PL	29	0.897 ± 0.096		OL vs OA	+0.024	0.223
		OA	104	0.900 ± 0.108		PL vs OA	-0.003	1.000
Hip	Female, 50-59	OL	694	0.875 ± 0.124	**0.335**	OL vs PL	+0.024	0.439
		PL	63	0.851 ± 0.116		OL vs OA	-0.001	1.000
		OA	131	0.876 ± 0.109		PL vs OA	-0.025	0.567
Hip	Female, 60-69	OL	1081	0.831 ± 0.126	**0.014**	OL vs PL	+0.038	**0.038**
		PL	73	0.793 ± 0.106		OL vs OA	+0.020	0.251
		OA	132	0.811 ± 0.122		PL vs OA	-0.018	0.986
Hip	Female, ≥70	OL	73	0.747 ± 0.130	**0.652**	OL vs PL	+0.017	1.000
		PL	16	0.730 ± 0.182		OL vs OA	-0.015	1.000
		OA	54	0.762 ± 0.110		PL vs OA	-0.032	1.000

Clusters derived from k-means (k = 3) on age/sex-residualized lifestyle variables (see **Supplementary Table 2**).

^a^Difference = Mean (Group1) – Mean (Group2).

^b^Using Bonferroni correction (correction factor=3).

Bold values indicate statistical significance.

OL, office/mental-labor workers with low leisure-time activity; PL, physical-labor workers; OA, office/mental-labor workers with an active leisure-time lifestyle. P-value from one-way ANOVA within each stratum; pairwise comparisons via Bonferroni-corrected *post-hoc* tests (correction factor = 3). SD, standard deviation.

#### 3.5.4 BMI stratification

Among participants with normal weight and overweight, the OA cluster showed significantly higher lumbar spine BMD than the OL cluster (differences of 0.034 and 0.041 g/cm^2^, both *P* < 0.001), and the PL cluster showed significantly lower hip BMD than the OL cluster in the overweight group (difference = −0.058, *P* < 0.001). Among obese participants, no significant pairwise differences in lumbar spine BMD were observed, although the PL cluster showed significantly lower hip BMD than the OL cluster (difference = −0.057, *P* = 0.041) (**Table 5**).

**Table 5:** Exploratory BMI-stratified BMD comparison across clusters.

Site	BMI group	Cluster	N	Mean ± SD (g/cm^2^)	*P*-value	Pairwise	Difference (g/cm^2^)^a^	Adjusted *P*^b^
Lumbar	Normal (<24)	OL	1481	0.879 ± 0.149	**<0.001**	OL vs PL	-0.002	1.000
		PL	146	0.881 ± 0.144		OL vs OA	-0.034	**<0.001**
		OA	369	0.913 ± 0.142		PL vs OA	-0.032	0.078
Lumbar	Overweight (24-27.9)	OL	1144	0.938 ± 0.151	**<0.001**	OL vs PL	-0.007	1.000
		PL	129	0.945 ± 0.234		OL vs OA	-0.041	**<0.001**
		OA	252	0.979 ± 0.142		PL vs OA	-0.034	0.155
Lumbar	Obese (≥28)	OL	391	0.974 ± 0.157	**0.354**	OL vs PL	-0.031	0.761
		PL	47	1.005 ± 0.239		OL vs OA	-0.023	0.926
		OA	69	0.997 ± 0.190		PL vs OA	+0.008	1.000
Hip	Normal (<24)	OL	1703	0.855 ± 0.135	**0.774**	OL vs PL	+0.009	1.000
		PL	136	0.846 ± 0.144		OL vs OA	0.000	1.000
		OA	378	0.855 ± 0.130		PL vs OA	-0.009	1.000
Hip	Overweight (24-27.9)	OL	1403	0.922 ± 0.134	**<0.001**	OL vs PL	+0.058	**<0.001**
		PL	128	0.864 ± 0.126		OL vs OA	+0.001	1.000
		OA	297	0.921 ± 0.135		PL vs OA	-0.057	**<0.001**
Hip	Obese (≥28)	OL	476	0.964 ± 0.148	**0.034**	OL vs PL	+0.057	**0.041**
		PL	43	0.907 ± 0.126		OL vs OA	+0.019	0.800
		OA	90	0.945 ± 0.142		PL vs OA	-0.038	0.456

Clusters derived from k-means (k = 3) on age/sex-residualized lifestyle variables (see **Supplementary Table 2**).

^a^Difference = Mean (Group1) – Mean (Group2).

^b^Using Bonferroni correction (correction factor=3).

Bold values indicate statistical significance.

OL, office/mental-labor workers with low leisure-time activity; PL, physical-labor workers; OA, office/mental-labor workers with an active leisure-time lifestyle. *P*-value from one-way ANOVA within each stratum; pairwise comparisons via Bonferroni-corrected *post-hoc* tests (correction factor = 3). SD, standard deviation.

### 3.6 Dose-response analyses of modifiable factors and BMD

In the full cohort, exercise and sun exposure showed significant non-linear associations with BMD at both skeletal sites (*P* for departure from linearity < 0.05; **Supplementary Table 7a**), while calcium supplementation showed a consistent linear negative association with no evidence of non-linearity (**Supplementary Table 7a**). Exercise showed the strongest, most consistent categorical association: relative to the lowest exercise category, the highest exercise category was associated with 0.082 g/cm^2^ higher lumbar spine BMD and 0.069 g/cm^2^ higher hip BMD (both *P* < 0.001; **Supplementary Table 7b**). Sun exposure showed a similar categorical pattern for hip BMD (0.040–0.062 g/cm^2^ higher across categories relative to the lowest, all *P* < 0.001; **Supplementary Table 7b**), while its relationship with lumbar spine BMD was less consistent, potentially reflecting the ambiguity of the “not noticed” response category. Calcium supplementation showed a monotonic negative linear association with BMD at both sites (lumbar spine β = −0.010, *P* = 0.003; hip β = −0.014, *P* < 0.001; **Supplementary Table 7a**). Model fit statistics and residual diagnostics for these models are reported in **Supplementary Table 7c**.

### 3.7 Interaction effects analysis

Cluster × exposure interaction analyses, estimated in the full cohort rather than conditioned within clusters (**Table 6**), showed that exercise had the most consistent effect modification by cluster at both skeletal sites (*P* < 0.001 for lumbar spine, *P* = 0.047 for hip). The exercise–BMD slope was steepest in the OL cluster (lumbar spine β = 0.048; hip β = 0.047) and markedly attenuated in the PL and OA clusters (lumbar spine β ≈ 0.000 and 0.014, respectively; hip β = 0.021 and 0.018, respectively), consistent with diminishing marginal returns of additional exercise among individuals with already-higher baseline activity levels. Sun exposure also showed significant effect modification (*P* = 0.032 for lumbar spine, *P* = 0.021 for hip), although the pattern was less consistent across clusters. Calcium supplementation showed no evidence of effect modification by cluster at either site (*P* = 0.532 and *P* = 0.831, respectively), reinforcing the interpretation that its negative association with BMD is independent of lifestyle cluster and more consistent with reverse causation than a cluster-specific behavioral effect. Because the cluster variable was itself partly derived from the exposure variables examined here, residual collinearity may remain in these interaction estimates; this analysis should be interpreted as a sensitivity analysis rather than a fully independent test of effect modification.

**Table 6:** Exploratory cluster × exposure interaction analyses in the full cohort (age/sex-residualized K-means clusters).

Outcome	Exposure	N	Cluster × exposure interaction *p*	Cluster-specific slope (linear-coded, β)	Interpretation
Lumbar spine	Sun exposure	4028	**0.032**	OL: 0.0072 (*P* = 0.270) PL: -0.0178 (*P* = 0.011 for Δ vs OL) OA: 0.0135 (*P* = 0.473 for Δ vs OL)	Association differs significantly across clusters (effect modification present)
Lumbar spine	Exercise time	4028	**<0.001**	OL: 0.0484 (*P*=<0.001) PL: 0.0004 (*P*=<0.001 for Δ vs OL) OA: 0.0140 (*P*=<0.001 for Δ vs OL)	Association differs significantly across clusters (effect modification present)
Lumbar spine	Calcium supplementation	4028	0.532	OL: -0.0144 (*P*=<0.001) PL: -0.0022 (*P* = 0.272 for Δ vs OL) OA: -0.0019 (*P* = 0.150 for Δ vs OL)	No evidence of effect modification by cluster
Hip	Sun exposure	4654	**0.021**	OL: 0.0240 (*P*=<0.001) PL: 0.0003 (*P* = 0.003 for Δ vs OL) OA: 0.0013 (*P* = 0.001 for Δ vs OL)	Association differs significantly across clusters (effect modification present)
Hip	Exercise time	4654	**0.047**	OL: 0.0466 (*P*=<0.001) PL: 0.0211 (*P*=<0.001 for Δ vs OL) OA: 0.0183 (*P*=<0.001 for Δ vs OL)	Association differs significantly across clusters (effect modification present)
Hip	Calcium supplementation	4654	0.831	OL: -0.0130 (*P*=<0.001) PL: -0.0229 (*P* = 0.305 for Δ vs OL) OA: -0.0126 (*P* = 0.953 for Δ vs OL)	No evidence of effect modification by cluster

Models fit in the full analytic sample (not conditioned within clusters), adjusted for age, sex, BMI, and hospital. Interaction *P*-value derived from a nested F-test comparing a model with cluster × exposure (categorical) interaction terms against a main-effects-only model. Cluster-specific slopes are from a parallel model coding exposure as a linear term interacted with cluster, shown for interpretability. OL, office/mental-labor workers with low leisure-time activity (reference; n = 3,803, 71.5%); PL, physical-labor workers (n = 453, 8.5%); OA, office/mental-labor workers with an active leisure-time lifestyle (n = 1,068, 20.0%). Exercise showed the most consistent effect modification: the exercise–BMD slope was steepest in the OL (reference) group at both skeletal sites and attenuated in the PL and OA groups, consistent with diminishing marginal returns of additional activity among those with already-higher baseline activity levels. Calcium supplementation showed no evidence of effect modification by cluster at either site, consistent with a cluster-independent (e.g., reverse-causation) association. BMD, bone mineral density.

Bold values indicate statistical significance.

## 4 Discussion

This study found that BMD differed significantly across the three revised lifestyle clusters. After adjustment for age (modeled flexibly), sex, BMI, and hospital, the OA cluster showed higher adjusted lumbar spine BMD than the OL cluster (adjusted difference = 0.024 g/cm^2^, 95% CI 0.012–0.036, *P* < 0.001), and the PL cluster showed lower adjusted hip BMD than both the OL cluster (adjusted difference = −0.041 g/cm^2^, 95% CI −0.056 to −0.027, *P* < 0.001) and the OA cluster (adjusted difference = 0.028 g/cm^2^, 95% CI 0.011–0.044, *P* = 0.002). These associations are broadly consistent with prior evidence linking physical activity to skeletal health. A randomized controlled trial in middle-aged and elderly men (≥45 years) reported that eight months of high-intensity resistance and impact training was associated with significantly higher BMD compared with a control group (11). A study of male long-distance runners found that those who additionally performed resistance training at least once weekly had higher BMD than peers who did not (12). A meta-analysis reported that exercise was associated with higher BMD in postmenopausal women with osteoporosis (13). Mechanistically, aerobic and weight-bearing exercise is thought to promote osteoblast activity and inhibit osteoclast differentiation through mechanical load stimulation, thereby supporting bone matrix deposition and trabecular microarchitecture (13–15); in animal models, incremental weight-bearing load has been associated with improved trabecular microarchitecture and preserved cortical bone properties (14), and mechanical loading may reduce sclerostin-mediated inhibition of the Wnt signaling pathway, thereby supporting bone formation (15). Sunlight exposure is similarly thought to support bone metabolism via cutaneous vitamin D synthesis and enhanced intestinal calcium absorption (16). Notably, ANOVA contribution analysis demonstrated that calcium supplementation contributed negligibly to cluster differentiation (η^2^ = 0.0006, *P* = 0.195; **Supplementary Table 3**), yet the OA cluster exhibited the most favorable lumbar spine BMD profile. This suggests that, provided baseline nutritional needs are met, active leisure-time exercise and sun exposure may offer bone health benefits independent of calcium supplementation alone.A 24-week study of elderly individuals with osteoporosis similarly found that a daytime brisk-walking regimen was associated with greater BMD gains than either a nighttime walking or a sunlight-only regimen (17), broadly consistent with our observations.

The finding that the PL cluster showed lower adjusted hip BMD than both the OL cluster (adjusted difference = −0.041 g/cm^2^, 95% CI −0.056 to −0.027, *P* < 0.001) and the OA cluster (adjusted difference = 0.028 g/cm^2^, 95% CI 0.011–0.044, *P* = 0.002) may appear counterintuitive given the occupational physical activity inherent to manual labor, but is consistent with the principle that bone’s adaptive response to mechanical loading depends on the pattern of loading (magnitude, rate, and directional variability) rather than on total activity volume alone. Occupational manual labor typically involves repetitive, sustained, and often uniaxial loading patterns; bone cells are thought to become progressively less responsive to a habitual, unvarying loading stimulus, producing diminishing osteogenic returns over time (18). In contrast, the high-impact, high-rate, and multidirectional loading characteristic of deliberate leisure-time exercise (e.g., jogging, jumping, resistance training)—more prevalent in the OA cluster—may provide a more potent osteogenic stimulus at the hip specifically, a site known to be particularly responsive to dynamic, high-magnitude loading. We were unable to further examine this hypothesis in the present dataset, as our exercise variable captured self-reported duration rather than the type, intensity, or biomechanical pattern of activity; this represents an important direction for future research incorporating objectively measured physical activity.

The magnitude of these adjusted associations warrants cautious interpretation. The observed differences (approximately 0.014–0.041 g/cm^2^ across comparisons) are statistically robust but numerically modest relative to the overall range of BMD values in this cohort, and considerably smaller than the unadjusted differences reported in cross-sectional comparisons of more extreme lifestyle categories in some prior literature. Established thresholds linking a specific BMD difference of this magnitude to a defined change in fracture risk are not well established, and the clinical significance of these associations—as distinct from their statistical significance—remains uncertain. We therefore present these findings as evidence of a consistent, biologically plausible pattern of association rather than as evidence of a clinically substantial protective effect, and we caution against over-interpreting the absolute magnitude of these differences in terms of fracture-risk reduction.

Stratified analyses by sex and age group showed a generally consistent pattern, with the OA cluster tending toward higher BMD and the PL cluster toward lower hip BMD across most strata, although these stratified comparisons are exploratory and subject to reduced statistical power within smaller subgroups. Notably, no significant lumbar spine differences among clusters were observed in women aged 50–59, a period coinciding with the perimenopausal transition. Estrogen decline in this period is well established as a determinant of accelerated bone loss; estrogen inhibits osteoblast apoptosis and osteoclast formation and is a key regulator of bone metabolism (19–21), and its decline is associated with increased osteoclastogenesis, prolonged osteoclast lifespan, elevated bone turnover, and accelerated bone resorption (22). It is plausible that the abrupt, estrogen-driven component of bone loss during this period attenuates the relative contribution of lifestyle factors to lumbar BMD, underscoring that perimenopausal and postmenopausal women may benefit from a comprehensive management strategy encompassing physical activity, sun exposure, and adequate calcium intake rather than any single modifiable behavior in isolation.

Among participants aged ≥70 years, no significant difference in hip BMD was observed among the three clusters; however, a significant pairwise difference in lumbar spine BMD persisted between the PL and OA clusters among women in this age group, suggesting that lifestyle-related differences in lumbar BMD may persist into advanced age even as hip-BMD differences attenuate. The broader attenuation of lifestyle-associated differences in this age group is consistent with prior literature (23): aging is associated with increased inflammation, oxidative stress, vascular calcification, and reduced autophagy, all of which may impair bone formation and accelerate bone loss (24), and age-related decline in Wnt/β-catenin signaling activity may further inhibit osteoblast differentiation and bone formation (25). We speculate that the relative contribution of lifestyle factors to BMD may diminish with advancing age, as genetic predisposition and intrinsic aging processes become increasingly dominant determinants (26).

BMI-stratified analyses showed that the association between cluster and lumbar spine BMD was present among participants with normal weight and overweight but not among those with obesity, while the association between cluster and hip BMD was, conversely, more evident among overweight and obese participants than among those with normal weight. The attenuated lumbar association among obese participants may relate to the chronic low-grade inflammatory state characteristic of obesity, in which continuous osteoclast activation and adipose-derived inflammatory cytokines (e.g., TNF-α, IL-6) may disrupt bone metabolic balance and blunt the skeletal benefits otherwise associated with favorable lifestyle patterns (27–29); sequestration of vitamin D in adipose tissue, reducing its bioavailability, may represent an additional contributing mechanism (30).

In dose-response and interaction analyses conducted in the full cohort, sun exposure showed a negative association with hip BMD specifically within the PL cluster in earlier, cluster-conditioned analyses. We interpret this negative within-cluster association with caution: because cluster membership was itself partly derived from the sun-exposure variable, conditioning on cluster membership can induce collider-type distortions in the relationships among clustering variables, providing a more parsimonious explanation for this pattern than a true biological effect of excessive ultraviolet exposure. Exercise showed the most consistent effect modification by cluster, with the steepest exercise–BMD slope observed in the OL cluster and progressively attenuated slopes in the PL and OA clusters, consistent with diminishing marginal returns of additional exercise among individuals with already-higher baseline activity levels. Calcium supplementation frequency, by contrast, showed a consistent negative association with BMD that did not differ significantly by cluster (interaction *P* = 0.532 for lumbar spine and *P* = 0.831 for hip; **Table 6**). This lack of effect modification—observed uniformly across clusters with markedly different overall activity profiles—argues against a cluster-specific behavioral mechanism underlying this association, and is more consistent with reverse causation, whereby individuals with lower BMD may be more inclined to initiate or intensify calcium supplementation, than with a true adverse or neutral effect of supplementation itself. Nonetheless, given the potential risks associated with long-term excessive calcium supplementation, such as vascular calcification, this warrants further investigation, ideally using longitudinal designs capable of establishing temporal precedence. We emphasize that our cross-sectional design cannot definitively distinguish reverse causation from a true adverse effect of calcium supplementation; the interaction analysis provides evidence more consistent with the former, but longitudinal studies establishing temporal precedence are needed to resolve this question definitively.

More broadly, these findings should be considered within the context of a growing literature on the combined, multidimensional influence of diet and lifestyle on musculoskeletal health through oxidative-stress-related pathways. A recent population-based study using a composite oxidative balance score integrating dietary and lifestyle pro- and anti-oxidant exposures reported a significant inverse association with sarcopenia and osteosarcopenia (31). Notably, that same study found no significant association between this oxidative balance score and low BMD considered in isolation, suggesting that the influence of oxidative stress may be more directly relevant to muscle than to bone tissue. This distinction cautions against over-attributing the lifestyle-BMD associations observed in the present study to a unified oxidative-stress mechanism, and underscores that the pathways linking lifestyle to bone versus muscle health may only partially overlap.

This study has several limitations. First, the cross-sectional design cannot establish causal relationships and should be considered exploratory; although we observed associations between lifestyle clusters and BMD, reverse causation cannot be fully excluded. Second, although the overall sample size was relatively large, only 26.7% of the initial cohort had complete lifestyle-questionnaire data. A substantial proportion of participants lacked complete lifestyle-questionnaire data, predominantly due to structural non-collection at two of the four participating hospitals rather than random individual non-response. We did not apply multiple imputation to the lifestyle variables themselves, both because the missingness mechanism at these two hospitals does not satisfy the missing-at-random assumption, and because imputing variables that were subsequently used to derive lifestyle clusters would artificially reduce the between-individual variability the clustering was intended to capture. Compared with the overall age-eligible population, the analytic sample was comparable in BMI and hip BMD, but skewed toward older and predominantly female participants, likely reflecting between-hospital differences in patient case-mix; this may limit the generalizability of the lifestyle-clustering results to the full source population. Third, the lifestyle data were based on self-report; although quality-control procedures were implemented, recall bias and social desirability bias cannot be excluded. The questionnaire items, while developed through a structured Delphi process establishing content relevance, were not subjected to formal reliability or construct-validity testing, and we did not record detailed indicators such as dietary calcium intake or vitamin D status. We also note that the exercise item captured self-reported daily duration rather than intensity; these are related but distinct constructs, and our findings should not be interpreted as characterizing exercise intensity specifically. Fourth, because the cluster variable was itself partly derived from the exposure variables examined in the interaction analysis, residual collinearity may remain in the cluster × exposure interaction estimates; this analysis should be interpreted as a sensitivity analysis rather than a fully independent test of effect modification. Fifth, DXA device type, menopausal status, relevant diseases and medications, dietary calcium intake, vitamin D status, examination season, and socioeconomic factors were not systematically available in our database and could not be included as covariates; hospital was included as a fixed effect, which may partially capture unmeasured between-center differences. Relatedly, we cannot exclude the possibility that unmeasured comorbidities independently influence both BMD and calcium-supplementation behavior—for example, individuals with underlying conditions affecting bone metabolism may be more likely both to have lower BMD and to supplement calcium more frequently—which would represent a further source of confounding beyond reverse causation alone in the calcium-BMD association. Additionally, DXA measurements were obtained using two different device manufacturers (Hologic Horizon-Wi at Hospitals 1–3; GE Lunar iDXA at Hospital 4), and no formal cross-calibration was performed between hospitals or devices; however, the analytic sample used for the primary lifestyle-cluster analyses was drawn entirely from Hospitals 1 and 2, both using Hologic Horizon-Wi, so cross-manufacturer comparability does not affect the core findings, and this limitation is more relevant to the overall-population descriptive statistics (**Supplementary Table 4**), which span all four hospitals. Finally, all study participants were drawn from a health-examination population across a relatively small number of centers, which may limit the generalizability of these findings to more diverse populations; the results should therefore be interpreted with appropriate caution.

In summary, this cluster-based analysis identified a lifestyle pattern combining active leisure-time behavior with office-based occupation as associated with more favorable lumbar spine BMD, and a physical-labor lifestyle as associated with lower hip BMD relative to both other clusters, despite the occupational physical activity inherent to manual labor. These associations exhibited site-specific patterns—spine BMD tracking more closely with leisure-time exercise and sun exposure, and hip BMD showing a distinct relationship with occupational category—that were broadly consistent across demographic strata, though attenuated among individuals aged ≥70 years and those with obesity. These findings suggest that occupational physical activity and deliberate leisure-time exercise may not be interchangeable with respect to their association with skeletal health at different anatomical sites, and that lifestyle-based interventions for bone health may benefit from consideration of both activity type and site-specific outcomes. Prospective studies are needed to confirm these associations and clarify their causal structure.

## Data Availability

The raw data supporting the conclusions of this article will be made available by the authors, without undue reservation.
