# Supplementary material for: Lifestyle clusters and bone mineral density in Chinese adults: a cross-sectional study with cluster analysis
Source: Front Endocrinol (Lausanne). 2026 Sep 4;17:1904158. doi: 10.3389/fendo.2026.1904158 (PMC13585520; doi:10.3389/fendo.2026.1904158)
Supplement: Supplementary file 1 [file SupplementaryFile1.docx]

Supplementary Material

# 1 Supplementary Text

**Supplementary Text 1.** Justification for retaining k = 3 over k = 4 in the age/sex-residualized K-means clustering

Cluster-selection diagnostics (Supplementary Figure 1) showed k = 4 had a marginally higher point-estimate silhouette width than k = 3 (0.315 vs. 0.309) and a comparable Calinski-Harabasz index (879.98 vs. 881.08); both solutions were similarly stable across independent multi-start searches (pairwise Adjusted Rand Index ≥ 0.96 for k = 4, ≥ 0.99 for k = 3). To determine whether the small silhouette advantage of k = 4 reflected a genuine, reproducible improvement in fit rather than sampling noise, we performed a bootstrap comparison (50 resamples with replacement, re-clustering at each resample). The bootstrap 95% confidence interval for the difference in silhouette width (k = 4 minus k = 3) was −0.105 to 0.083, comfortably spanning zero; k = 3 exceeded k = 4 in exactly 50% of resamples. This indicates the apparent advantage of k = 4 is not statistically distinguishable from sampling noise. Given the absence of reliable evidence favoring the additional complexity of a fourth cluster, k = 3 was retained as the primary solution following the principle of parsimony.

# 2 Supplementary Figures and Tables

## 2.1 Supplementary Figures

**Supplementary Figure 1.** Cluster-selection diagnostics for age/sex-residualized k-means clustering, k = 2–6.

Each of the eight lifestyle variables was first residualized on age and sex (logistic regression for binary items; proportional-odds ordinal logistic regression for ordinal items), then standardized prior to clustering. Silhouette width (left) and Calinski-Harabasz index (right) were computed using the optimized multi-start procedure. The red point marks the adopted k = 3 solution (silhouette width = 0.309, Calinski-Harabasz index = 881.1), exceeding the conventional silhouette threshold of 0.25. Although k = 4 showed a marginally higher point-estimate silhouette width (0.315), a bootstrap comparison (50 resamples) showed this difference was not distinguishable from sampling noise (95% CI for the difference: −0.105 to 0.083), supporting retention of k = 3. N = 5,324.

## 2.2 Supplementary Tables

**Supplementary Table 1.** Distribution of lifestyle variables in the clustered sample (N = 5,324).

| **Lifestyle Variable** | **Category** | **Percentage (n)** |
| --- | --- | --- |
| Self-rated health | Average | 89.4% (n=4,758) |
|  | Healthy | 10.6% (n=566) |
| Occupation type | Mental labor | 91.5% (n=4,871) |
|  | Physical labor | 8.5% (n=453) |
| Daily sun exposure | Not noticed | 6.9% (n=368) |
|  | <30 min | 64.6% (n=3,441) |
|  | 30–60 min | 15.9% (n=849) |
|  | >60 min | 12.5% (n=666) |
| Daily exercise time | Rarely/almost no exercise | 12.5% (n=663) |
|  | <30 min | 34.1% (n=1,817) |
|  | 30–60 min | 39.8% (n=2,121) |
|  | >60 min | 13.6% (n=723) |
| Smoking | Yes | 16.7% (n=891) |
|  | No | 83.3% (n=4,433) |
| Alcohol consumption | Frequent/heavy | 2.7% (n=146) |
|  | Occasional/light | 9.3% (n=495) |
|  | None | 88.0% (n=4,683) |
| Daily beverage preference | Coffee/carbonated drinks | 5.2% (n=279) |
|  | Water/juice/other | 9.4% (n=502) |
|  | Milk/tea | 85.3% (n=4,543) |
| Calcium supplementation | None | 57.3% (n=3,051) |
|  | Occasional | 28.6% (n=1,520) |
|  | Regular/long-term | 14.1% (n=753) |

**Supplementary Table 2.** Stability verification of the age/sex-residualized K-means clustering (k = 3).

| **Outer seed** | **Cluster sizes** | **Silhouette width** | **Pairwise ARI vs. other seeds** |
| --- | --- | --- | --- |
| 1 | 3804 / 453 / 1067 | 0.3091 | 0.992-1.000 |
| 7 | 3812 / 453 / 1059 | 0.3093 |  |
| 42 | 3803 / 453 / 1068 | 0.3091 |  |
| 99 | 3803 / 1068 / 453 | 0.3091 |  |
| 123 | 1068 / 3803 / 453 | 0.3091 |  |

Note: N = 5,324. Five independent optimized searches (different outer random seeds, 50 k-means++ restarts each, best solution retained by inertia) converged to essentially the same partition (pairwise Adjusted Rand Index ≥ 0.99). Silhouette width for the adopted k = 3 solution was 0.309, exceeding the conventional threshold of 0.25 for meaningful cluster structure. Justification for retaining k = 3 over k = 4 is provided in Supplementary Text 1. OL, office/mental-labor workers with low leisure-time activity; PL, physical-labor workers; OA, office/mental-labor workers with an active leisure-time lifestyle.

**Supplementary Table 3.** Between-cluster explanatory power (η²) of each lifestyle variable in the final cluster solution

| **Lifestyle Variable** | **η² (Between-Cluster Explanatory Power)** | ***P*** |
| --- | --- | --- |
| Occupation type | 0.9999 | <0.001 |
| Sun exposure | 0.3034 | <0.001 |
| Beverage preference | 0.2797 | <0.001 |
| Self-rated health | 0.2424 | <0.001 |
| Exercise time | 0.1488 | <0.001 |
| Alcohol consumption | 0.0125 | <0.001 |
| Smoking | 0.0030 | <0.001 |
| Calcium supplementation | 0.0006 | 0.195 |

η² = between-cluster sum of squares / total sum of squares, computed from a one-way ANOVA of each age/sex-residualized lifestyle variable on the final k = 3 cluster assignment (N = 5,324). Higher values indicate greater contribution to cluster separation.

**Supplementary Table 4.** Baseline characteristics of the overall population.

| Variable | N | Mean or count | SD or % |
| --- | --- | --- | --- |
| Age | 19,934 | 54.52 | 14.47 |
| Male | 19,934 | 9865 | 49.50% |
| Height (cm) | 12,647 | 164.63 | 7.85 |
| Weight (kg) | 12,646 | 66.05 | 11.77 |
| BMI (kg/m²) | 12,645 | 24.29 | 3.44 |
| Lumbar BMD | 16,913 | 0.93 | 0.16 |
| Hip BMD | 18,127 | 0.9 | 0.15 |

BMD, bone mineral density; SD, standard deviation.

**Supplementary Table 5.** Representativeness of the analytic sample relative to the overall age-eligible population.

| **Characteristic** | **Analytic sample (complete questionnaire) (N = 5,324)** | **Overall age-eligible population (N = 19,934)** | ***P*ᵃ** |
| --- | --- | --- | --- |
| Age, years, mean ± SD | 56.2 ± 11.8 | 54.5 ± 14.5 | **<0.001** |
| Female sex, n (%) | 3291 (61.8%) | 10069 (50.5%) | **<0.001** |
| BMI, kg/m², mean ± SD | 24.31 ± 3.37 | 24.29 ± 3.44 | 0.579 |
| Lumbar spine BMD, g/cm², mean ± SD (n available) | 0.92 ± 0.159 (n = 4031) | 0.928 ± 0.159 (n = 16913) | **<0.001** |
| Hip BMD, g/cm², mean ± SD (n available) | 0.893 ± 0.142 (n = 4654) | 0.896 ± 0.146 (n = 18127) | 0.097 |

ᵃ *P*-values compare the analytic sample (participants with complete 8-item lifestyle questionnaire data, used for clustering) against the remaining age-eligible participants without complete questionnaire data, using independent-samples t-test (age, BMI, BMD) or chi-square test (sex); this provides a valid significance test while the descriptive columns show the analytic sample alongside the overall age-eligible population (N = 19,934) from which it was drawn, to assess representativeness. The analytic sample was comparable to the overall population in BMI and hip BMD; it was significantly older and included a higher proportion of women, and showed a statistically significant but numerically trivial difference in lumbar spine BMD (a 0.008 g/cm² difference). This demographic skew likely reflects between-hospital differences in patient case-mix that also underlie the differential questionnaire completion rates across hospitals (see Figure 1), and is noted as a limitation regarding the generalizability of the lifestyle-clustering results to the full source population. BMD, bone mineral density; BMI, body mass index; SD, standard deviation.

**Supplementary Table 6.** BMD comparison across three lifestyle clusters (age/sex-residualized K-means, k = 3).

| **Site** | **Cluster** | **N** | **Mean ± SD (g/cm²)** | ***P*-value** | **Pairwise** | **Difference** | **Adjusted *P*** |
| --- | --- | --- | --- | --- | --- | --- | --- |
| **Lumbar** | OL | 3016 | 0.914 ± 0.155 | **<0.001** | PL vs OL | 0.011 | 0.751 |
|  | PL | 323 | 0.925 ± 0.203 |  | OA vs OL | 0.032 | **<0.001** |
|  | OA | 692 | 0.946 ± 0.152 |  | OA vs PL | 0.021 | 0.139 |
| **Hip** | OL | 3582 | 0.896 ± 0.143 | **<0.001** | PL vs OL | -0.034 | **<0.001** |
|  | PL | 307 | 0.862 ± 0.135 |  | OA vs OL | -0.004 | 1.000 |
|  | OA | 765 | 0.891 ± 0.138 |  | OA vs PL | 0.029 | **0.007** |

Note: Clusters derived from k-means (k = 3) applied to the eight lifestyle variables after residualizing each on age and sex; solution verified stable across 5 independent multi-start searches (pairwise Adjusted Rand Index ≥ 0.99). OL, office/mental-labor workers with low leisure-time activity (n = 3,803, 71.5%); PL, physical-labor workers (n = 453, 8.5%); OA, office/mental-labor workers with an active leisure-time lifestyle (n = 1,068, 20.0%). Overall P from one-way ANOVA; pairwise *P* from Bonferroni-corrected post-hoc comparisons (correction factor = 3). Unadjusted comparisons shown here; ANCOVA-adjusted comparisons (age, sex, BMI, hospital) are reported in Table 1. SD, standard deviation.

**Supplementary Table 7.** Exploratory dose-response analyses of modifiable lifestyle factors and BMD in the full cohort (categorical vs. linear specification).

**Supplementary Table 7a.** Linear coefficients and test for departure from linearity.

| **Outcome** | **Exposure** | **N** | **Linear β (95% CI)** | **Linear *P*** | ***P* for departure from linearity** |
| --- | --- | --- | --- | --- | --- |
| Lumbar spine BMD | Sun exposure | 4028 | 0.009 (0.003, 0.015) | 0.006 | **0.014** |
| Lumbar spine BMD | Exercise time | 4028 | 0.033 (0.027, 0.038) | <0.001 | **<0.001** |
| Lumbar spine BMD | Calcium supplementation | 4028 | -0.010 (-0.017, -0.003) | 0.003 | 0.197 |
| Hip BMD | Sun exposure | 4654 | -0.000 (-0.005, 0.004) | 0.847 | **<0.001** |
| Hip BMD | Exercise time | 4654 | 0.028 (0.024, 0.032) | <0.001 | **<0.001** |
| Hip BMD | Calcium supplementation | 4654 | -0.014 (-0.019, -0.009) | <0.001 | 0.755 |

BMD, bone mineral density

**Supplementary Table 7b.** Category-specific coefficients (relative to reference level).

| **Outcome** | **Exposure** | **Category (ref. level)** | **β (95% CI) vs reference** | ***P*** |
| --- | --- | --- | --- | --- |
| Lumbar spine BMD | Sun exposure | *ref = undefined* |  |  |
|  |  | 1 | -0.015 (-0.035, 0.004) | 0.115 |
|  |  | 2 | 0.006 (-0.016, 0.029) | 0.594 |
|  |  | 3 | 0.006 (-0.017, 0.030) | 0.607 |
| Lumbar spine BMD | Exercise time | *ref = undefined* |  |  |
|  |  | 1 | 0.036 (0.022, 0.050) | **<0.001** |
|  |  | 2 | 0.083 (0.069, 0.097) | **<0.001** |
|  |  | 3 | 0.082 (0.064, 0.100) | **<0.001** |
| Lumbar spine BMD | Calcium supplementation | *ref = undefined* |  |  |
|  |  | 1 | -0.016 (-0.026, -0.005) | **0.004** |
|  |  | 2 | -0.016 (-0.031, -0.001) | **0.034** |
| Hip BMD | Sun exposure | *ref = undefined* |  |  |
|  |  | 1 | 0.062 (0.046, 0.079) | **<0.001** |
|  |  | 2 | 0.060 (0.041, 0.078) | **<0.001** |
|  |  | 3 | 0.040 (0.021, 0.060) | **<0.001** |
| Hip BMD | Exercise time | *ref = undefined* |  |  |
|  |  | 1 | 0.056 (0.044, 0.067) | **<0.001** |
|  |  | 2 | 0.095 (0.084, 0.107) | **<0.001** |
|  |  | 3 | 0.069 (0.054, 0.083) | **<0.001** |
| Hip BMD | Calcium supplementation | *ref = undefined* |  |  |
|  |  | 1 | -0.015 (-0.023, -0.007) | **<0.001** |
|  |  | 2 | -0.027 (-0.038, -0.016) | **<0.001** |

Note: All models adjusted for age, sex, BMI, and hospital (fixed effect). "*P* for departure from linearity" (Supplementary Table 7a) derived from nested F-test (categorical model vs. linear-coded model). For sun exposure (daylight), category "Not noticed" reflects uncertain recall rather than greater duration; this variable is therefore not a strictly ordered duration scale and non-linear/threshold results should be interpreted with this measurement limitation in mind. BMD, bone mineral density; CI, confidence interval.

**Supplementary Table 7c.** Model fit and diagnostic information (categorical models).

| **Outcome** | **Exposure** | **N** | **R²** | **Adjusted R²** | **Model F-test *P*** | **Jarque-Bera normality *P*** |
| --- | --- | --- | --- | --- | --- | --- |
| Lumbar spine BMD | Sun exposure | 4028 | 0.153 | 0.152 | <0.001 | <0.001 |
| Lumbar spine BMD | Exercise time | 4028 | 0.185 | 0.183 | <0.001 | <0.001 |
| Lumbar spine BMD | Calcium supplementation | 4028 | 0.152 | 0.151 | <0.001 | <0.001 |
| Hip BMD | Sun exposure | 4654 | 0.265 | 0.264 | <0.001 | <0.001 |
| Hip BMD | Exercise time | 4654 | 0.297 | 0.296 | <0.001 | <0.001 |
| Hip BMD | Calcium supplementation | 4654 | 0.259 | 0.258 | <0.001 | <0.001 |

Note: R² and adjusted R² are for the full categorical model (exposure category + age, sex, BMI, hospital). Model F-test *P*-value tests the overall model against an intercept-only model. Residual normality was assessed via the Jarque-Bera test; all models showed statistically significant departure from normality (*P* < 0.001), which is common with large sample sizes (N > 4,000) and does not materially affect the validity of coefficient estimates or standard errors under the Central Limit Theorem given the sample sizes here. No evidence of results being driven by influential/outlier observations was found (see main text sensitivity analysis excluding implausible BMD values). BMD, bone mineral density.
